# Prevalence of Intestinal Parasitic Infections and Associated Risk Factors among Pregnant Women Attending Prenatal Care in the Northwestern Ethiopia

**DOI:** 10.1155/2021/3387742

**Published:** 2021-12-23

**Authors:** Gebre Ayanaw Alula, Abaineh Munshea, Endalkachew Nibret

**Affiliations:** ^1^Department of Biology, College of Science, Bahir Dar University, Ethiopia; ^2^Institute of Biotechnology, Bahir Dar University, Ethiopia

## Abstract

Intestinal parasitic infections (IPIs) are the common health problems in developing countries with low socioeconomic and poor living conditions. IPIs affect millions of pregnant women worldwide and may lead to adverse maternal and fetal effects. The present study was aimed at determining the prevalence and associated risk factors of IPIs among pregnant women in Ethiopia. A hospital-based cross-sectional study involving 384 pregnant women was conducted from November 2018 to March 2019. Relevant information on potential risk factors associated with IPIs was gathered using a semistructured questionnaire. Stool samples were collected and examined using wet mount and formol-ether concentration techniques. Logistic regression was used to evaluate the possible association between dependent and independent variables. The overall prevalence of IPIs was 36.7%. Seven species of parasites were identified. The most prevalent intestinal protozoan parasite identified was *Entamoeba histolytica/dispar* (9.6%) followed by *Giardia intestinalis* (8.9%). The predominant helminth parasite identified was *Ascaris lumbricoides* (8.6%), followed by hookworm (5.2%), *Taenia* spp. (3.6%), *Strongyloides stercoralis* (1.3%), and *Schistosoma mansoni* (1.04%). Six pregnant women (1.56%) had infection by two parasite species. The odds of IPIs were higher among illiterates (AOR = 4.63), lowest monthly income earners (AOR = 3.49), primigravida (pregnant for the first time) (AOR = 2.04), those who used unboiled well/stream/river water for drinking (AOR = 14.55), ate soil (AOR = 2.32), and consumed raw vegetables (AOR = 1.91). The prevalence of IPIs in the study subjects was substantially high. Thus, screening of the women for IPIs and providing health education during their antenatal care (ANC) visit are recommended to prevent possible adverse maternal and fetal effects resulting from these infections.

## 1. Introduction

Intestinal parasitic infections (IPIs), caused by helminths and protozoan parasites, are among the most common public health problems in developing countries with low income, low level of education, lack of clean water supply, and poor personal and environmental sanitation [1, 2]. The most common intestinal protozoan parasites of humans are *Entamoeba histolytica/dispar*, *Giardia intestinalis*, and *Cryptosporidium* spp., whereas the most common parasitic helminths affecting humans are *Ascaris lumbricoides*, *Trichuris trichiura*, hookworm (*Ancylostoma duodenale* and *Necator americanus*), and *Strongyloides stercoralis* [3].

The majority of cases of IPIs are asymptomatic; however, they can cause a wide spectrum of clinical symptoms such as diarrhea, nausea, vomiting, dehydration, abdominal pain, fever, and bloating and weight loss [4]. The pathogenesis, morbidity, and mortality of intestinal parasites depend upon the species of the parasite and the age, gender, immunological status, and nutritional status of the host [5]. In children, IPIs may cause vitamin A deficiency, iron deficiency anemia, growth retardation, and poor educational performance [4]. IPIs can result in severe complications in immune compromised patients such as those with human immunodeficiency virus (HIV) [6], transplant recipients, and hemodialysis patients [7].

In Ethiopia, IPIs are the major causes of morbidity and mortality. They inflict serious public health problems such as malnutrition, anemia, and elevated susceptibility to other types of infections [8]. Ethiopia has one of the lowest quality drinking water supply and latrine coverage in the world, and because of this and other risk factors, IPIs are the second most predominant causes of outpatient morbidity in the country [9]. According to a report by the Ministry of Health (MOH) of Ethiopia, helminthiasis is the third leading cause of outpatient visits in health institutions between 2005 and 2006 [10]. High prevalence of parasitic infections in Ethiopia is due to unsafe and inadequate provision of water, unhygienic living conditions, absence of proper utilization of latrine, and walking bare footed [9, 11].

Pregnancy is a physiological state and is often thought to be associated with increased risk of infection. This could be due to changes in immune function, such as a reduction in T, B, and natural killer cells activity and an increase in dendritic cell activity [12–14]. IPIs affect millions of pregnant women worldwide, and these infections directly or indirectly lead to adverse maternal and fetal effects, including maternal anemia [15, 16], low pregnancy weight gain, poor fetal growth [17], low birth weight, prenatal mortality, preterm birth [16], and poor cognitive and gross motor outcomes in infants [18]. Parasitic infection could occur at any stage of the three trimesters, but infection during the first trimester is associated with more severe fetal and placental consequences. Furthermore, the infection becomes more severe in women who are pregnant for the first time (primiparous) [19].

Several studies demonstrated very high prevalence of IPIs in different parts of Ethiopia [20, 21]. However, there are still localities in the country, including the present study area, Alefa District, for which information was lacking. Therefore, the present study was aimed at determining the prevalence and associated risk factors of intestinal parasitic infection among pregnant women attending prenatal care at the Shahura Primary Hospital, northwest Ethiopia.

## 2. Materials and Methods

### 2.1. Description of the Study Area

**Figure 1:**
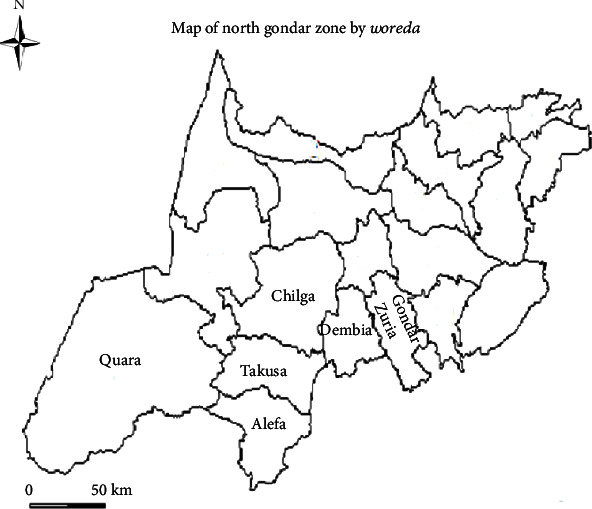
Map of the study area [22].

The study was conducted at Shahura Primary Hospital located in Alefa District, Shahura town (Figure 1). Shahura is the town of Alefa *Woreda* (district) in Amhara Region, North Gondar Zone, Ethiopia. It is bordered on the north by Takusa District, on the east by Lake Tana, on the west by Quara District, and on the southwest by West Gojjam Zone. Alefa district is found about 644 km from Addis Ababa to northwest and 142 km from Gondar town in the southwest. It is located at latitude of 12° 36′ N and longitude of 37° 28′ E with an elevation of 2133 m above sea level. Administratively, the district is divided into 37 *Kebeles* (the smallest administrative units in Ethiopia) with the total area of 189285 square kilometers [22].

Based on the Woreda Agricultural and Rural Development Office document, the district has two main agroclimatic conditions: *Woynadega* and *Qolla* accounting for 55% and 45%, respectively, of the total area. The average annual rainfall of the district ranges from 1800 to 2200 mm, and the average temperature ranges from 18° to 24°C [22].

The main economic activities of the rural population are mixed farming. Various types of crops and cereals, vegetables, and fruits grow in the district. Nearly 80% of the farmers are involved both in crop and livestock production while 15.4% grow crops only and 4.75% raise livestock only [22].

Based on the 2007 National Census conducted by Central Statistics Agency in Ethiopia, the population of Alefa District was 170491; among these, 86350 of them were males, and 84141 of them were females. Of the total population, 32559 were rural dwellers and engaged in agricultural activities [22]. The district has one Primary Hospital (Shahura Primary Hospital) and five health centers in five *Kebele*s of the town. This hospital serves 170491 people living in the district.

### 2.2. Study Design

A cross-sectional hospital-based study was conducted from November, 2018, to March, 2019, to determine the prevalence of intestinal parasitic infections and associated risk factors among pregnant women attending prenatal care at Shahura Primary Hospital, northwest Ethiopia.

### 2.3. Source and Study Population

#### 2.3.1. Source of Population

All pregnant women attending prenatal care at Shahura Primary Hospital were considered a source population for the study.

#### 2.3.2. Study Population

Pregnant women who were willing to participate and consented to provide stool samples and willing to provide information on sociodemographic and environmental sanitation were considered study population.

### 2.4. Inclusion and Exclusion Criteria

#### 2.4.1. Inclusion Criteria

Pregnant women at any gestational period who were willing to provide stool samples and to fill out questionnaire on sociodemographic and environmental sanitation related information at time of data collection were included in the study.

#### 2.4.2. Exclusion Criteria

Pregnant women who were taking antihelminthic/antiprotozoan drugs within the past two weeks and those who were seriously sick (unable to provide sociodemographic and environmental sanitary related information) at time of data collection were excluded from the study.

### 2.5. Sample Size Determination and Sampling Techniques

#### 2.5.1. Sample Size Determination

Sample size was determined using simple population proportion formula for sample size calculation [23]. Since the overall prevalence rate (*P*) of intestinal parasites among pregnant women in the study area was not known, prevalence was taken to be 50%. In the calculation, 95% confidence level (*z*) and 5% sampling error (*d*) were used.

(1) $$\begin{array}{c} n=Z^{2}P\;\left(1-p\right)/d^{2}, \end{array}$$

where *n* is the required sample size, *Z* is the confidence level at 95% (standard value of 1.96), *P* is the prevalence 50% (standard value of 0.5), *d* is the margin of error at 5% (standard value of 0.05), and *N* is (1.96)^2^0.5(1 − 0.5)/(0.05)^2^ = 384.

Therefore, a total of 384 pregnant women were included in the study.

#### 2.5.2. Sampling Techniques

A systematic random sampling technique was used to select the study participants. During the study, the average number of pregnant women attending prenatal care in the preceding three months was 785. This number was divided for the sample size to get the sample interval (*K*th = *N*/*n*) which was 2. Following the selection of the first study participants using the lottery method, every 2nd pregnant woman attending the hospital and who met the inclusion criteria was enrolled in the study until the calculated sample size was achieved.

### 2.6. Variables

#### 2.6.1. Independent Variable

Independent variables include sociodemographic and socioeconomic variables such as age, marital status, education level, residence, monthly income, and occupation, obstetric characteristics such as parity and gestational period, and potential risk factors like hand washing habit before meal and after defecation, toilet availability, place of bathing, shoes-wearing habit, source of drinking water, habit of eating soil, and unwashed fruit/vegetables/meat.

#### 2.6.2. Dependent Variable

*Dependent variables include* prevalence of intestinal parasitic infections among pregnant women attending prenatal care at Shahura Primary Hospital.

### 2.7. Methods of Data Collection

Questionnaire survey was conducted to assess the major sociodemographic and potential risk factors of intestinal parasitic infections among pregnant women. In addition, stool samples were collected to assess the prevalence of intestinal parasitic infections.

#### 2.7.1. Questionnaire Survey

A semistructured questionnaire was developed in English, and then the items of the questionnaire were translated into the local language, Amharic. The questionnaire included information on demographic characteristics (age, education, socioeconomic status, residence, data on potential contributing factors for intestinal parasitic infections, and obstetric information such as number of previous pregnancies and gestational age).

An explanation about the aim of the study was given to all voluntary participants by the principal investigator. After obtaining written consent from each of the voluntary participants, information about sociodemographic, potential risk factors of IPIs, and obstetric characteristics were gathered from 384 pregnant women. Obstetric information were recorded from the women's antenatal care (ANC) charts.

#### 2.7.2. Stool Sample Collection and Laboratory Procedures

After obtaining informed consent, orientation was given to the women on how to collect sufficient amount and contamination free stool specimens. Each study participant was provided with a labeled disposable plastic cup and applicator stick to bring about 3-4 g of stool. Then unique code of the pregnant woman was labeled on the cup, and then stool samples were processed for parasite species identification.

#### 2.7.3. Direct Wet Mount Method

About 2 mg of stool sample was emulsified with a drop of normal saline (0.85% NaCl solution). Then after, a drop of emulsified sample was placed on a slide, a few drops of iodine were added, and the slide was covered with cover slip. Each preparation was first examined under 10× objective lens, and then by 40× objective lens for specific identification of parasites [24].

#### 2.7.4. Formol-Ether Concentration Method

One gram (1 g) of stool specimen was placed in a centrifuge tube containing 7 ml of 10% formalin. The sample was suspended and mixed thoroughly with an applicator stick. The resulting suspension was filtered through a sieve (cotton gauze) into a beaker, and the filtrate was pour back into the same tube. Then after, 3 ml of diethyl ether was added to the mixture, and the tube was closed and shaken vigorously. Then, it was centrifuged at 1500 rpm for 2 minutes. After centrifugation, the supernatant (layers of ether, debris, and formalin) was discarded, and the sediment containing the parasites at the bottom of the test tube was resuspended. The sediment was then transferred to a slide using a Pasteur pipette and examined microscopically under 10× and 40× objective lenses for the presence of intestinal parasites [24].

### 2.8. Quality Control

The items of the questionnaire were adapted from questionnaires used in similar epidemiological studies conducted elsewhere with minor modifications to suit the local context and objective of the present study [8, 16, 17, 20, 21]. Before commencing the actual data collection, the questionnaire was pretested in 20 (5% of the total sample size) randomly selected pregnant women attending another health facility in the town to assess the clarity, appropriateness, and comprehensibility of the questionnaire. Then, misunderstandings identified in the items of the questionnaire were revised. Trained interviewers interviewed the participating women with their mother tongue “Amharic” to avoid confusion and ensure that the items of the questionnaire were clearly understood and interpreted as intended.

Standard operating procedures were followed for specimen collection and processing. To ensure reliability of the test procedures, the microscopes and reagents used in wet mount, and formol-ether concentration methods for detection of IPIs were also tested using known positive and negative stool samples as indicated in the stool sample collection and laboratory procedures. Stool samples were declared positive when various stages of the parasites, such as trophozoites, cysts, ova, and larvae, were observed. Besides, two laboratory technologists were employed to assist the microscopic identification of the parasites. To ensure general safety, disposable gloves were used, and universal biosafety precautions as per National Committee for Clinical Laboratory Standards (NCCLS) were followed at all times [25].

### 2.9. Data Analysis

Information recorded on the questionnaire and the results collected from laboratory were checked for completeness and consistency and then coded and entered into the computer. The compiled data were analyzed using Statistical Package for the Social Sciences (SPSS version 20). First, descriptive statistics were computed, and the result was reported using frequency and percentage. Logistic regression analysis was also used to measure the strength of association between potential risk factors with IPIs. A univariate logistic regression was first employed to select variables with a *p* value cut-off point of < 0.25 [26]. The selected variables in univariate analysis were entered into multivariate logistic regression model to identify the major explanatory variables of IPIs among studied pregnant women in the study area. Finally, variables in the final model of multivariate logistic regression with a *p* value of < 0.05 at 95% confidence interval were taken as statistically significant explanatory variables for IPIs in studied subjects.

## 3. Results

### 3.1. Sociodemographic and Obstetric Characteristics of Study Participants

A total of 384 pregnant women were included in the present study, and a 100% response rate was obtained in filling out the questionnaires and providing stool samples. The ages of participants ranged from 15 to 45 years, and the mean age (±SD) of the study participants was 25.7 ± 5.12 years. Majority of the study subjects were in the age group of 20-24 years (41.4%), married (74.2%), able to write and read (45.3%), urban dwellers (51.1%), earners of a monthly income between 500-1000 Ethiopian Birr (ETB) (48.4%), and housewives (69.8%) (Table 1).

### 3.2. The Overall and Species-Specific Prevalence of Intestinal Parasitic Infections

The overall prevalence of IPIs was 36.7% (141/384). Of this, the prevalence of intestinal protozoan parasites was 18.5% (71/384), and the prevalence of intestinal helminth parasites was 19.8% (76/384). In total, seven different species of intestinal parasites were identified, *E. histolytica/dispar* was the most prevalent protozoan parasite with a prevalence of 9.6% (37/384), followed by *G. intestinalis*, 8.9% (34/384). From helminthic parasites, *A. lumbricoides*, 8.6% (33/384), was the most prevalent followed by hookworm, 5.2% (20/384), *Taenia* species, 3.6% (14/384), *S. stercoralis*, 1.3% (5/384), and *S. mansoni*, 1.04% (4/384). High prevalence of each species was observed among age groups of 20-24 and 25-29 years whereas low prevalence was found among age group of 40 years and above.

### 3.3. Logistic Regression Analysis of Risk Factors Associated with IPIs

Logistic regression analysis was used to determine the degree of association and to identify the major explanatory risk factors of IPIs among pregnant women at Shahura Primary Hospital. A univariate logistic regression analysis was first done and then a *p* value with a cut-off point, 0.25 [27], was used for selecting the candidate variables for multivariable analysis to identify the major explanatory risk factors.

#### 3.3.1. Univariate and Multivariable Logistic Regression Analysis of Sociodemographic and Obstetric Risk Factors Potentially Associated with IPIs

The univariate logistic regression analysis of sociodemographic and obstetric characteristics associated with IPIs are presented as crude odds ratio (COR) at 95% CI in Table 2. There was no significant association between age, marital status, gravidity, and gestation period of pregnant women with that of IPIs (*p* > 0.05). Pregnant women, who were illiterate, those who could read and write, and those who attended high school were 5.69, 3.41, and 3.69 times (COR = 5.69, 95%CI = 2.25 − 14.42, *p* < 0.001; COR = 3.41, 95%CI = 1.44 − 8.04, *p* = 0.005; and COR = 3.69, 95%CI = 1.49 − 9.13, *p* = 0.005, respectively) more likely to be infected by IPIs than those having college and university education level, and the association is statistically significant (*p* < 0.05).

It was found that the risk of infection was 1.97 times (COR = 1.97, 95%CI = 1.29 − 3.00, *p* = 0.002) higher in rural pregnant women than in urban pregnant women. Similarly, about 3.77 times more likelihood of IPIs was observed among subjects whose monthly income was less than 500 Birr (ETB) (COR = 3.77, 95%CI = 2.06 − 6.91, *p* ≤ 0.001) compared to those whose monthly income was above 1000 Birr (ETB) (*p* < 0.05).

House wife pregnant women were 2.75 times (COR = 2.75, 95%CI = 1.27 − 5.95, *p* = 0.01) more likely to be infected by IPIs than civil servant pregnant women (*p* < 0.05). With regard to gravidity, those pregnant women with first gravida were 1.71 times (COR = 1.71, 95%CI = 0.88 − 3.31, *p* = 0.112) more likely to acquire IPIs compared to those multigravidae pregnant women; however, the association is not statistically significant (*p* > 0.05) (Table 2).

#### 3.3.2. Univariate and Multivariable Logistic Regression Analysis of Personal and Environmental Risk Factors Associated with IPIs

Univariate logistic regression analysis of potential risk factors associated with intestinal parasitic infections is shown in Table 3. It was found that the risk of infection was 2.39 times (COR = 2.39, 95%CI = 1.48 − 3.88, *p* ≤ 0.001) higher in pregnant women who did not wash their hands after defecation than those who washed their hands after defecation. Similarly the likelihood of IPIs was 4.35 times (COR = 4.35, 95%CI = 1.63 − 11.60, *p* < 0.001) higher in pregnant women who did not wash their hands before eating food than those who washed their hands before meal (*p* < 0.05).

Pregnant women who did not use latrine were 1.69 times (COR = 1.69, 95%CI = 1.09 − 2.63, *p* = 0.018) more likely to be infected by IPIs compared with those who used toilet (*p* < 0.05). Similarly, the likelihood of IPIs was almost 3 times (COR = 2.76, 95%CI = 1.49 − 5.12, *p* = 0.001) higher in pregnant women who did not wear shoes compared to those who wore shoes. It was also found that the risk of IPIs was 9.5 times (COR = 9.50, 95%CI = 1.17 − 76.88, *p* = 0.035) higher in pregnant women who used well/river/stream waters for drinking than those who used boiled water. The associations in terms of shoes wearing and source of drinking water with that of IPIs were statistically significant (*p* < 0.05).

Pregnant women who had habit of eating soil was 2.73 times (COR = 2.73, 95%CI = 1.41 − 5.28, *p* = 0.003) more likely to be infected by IPIs than those who did not consume soil. Similarly, the risk of infection was 2.58 times (COR = 2.58, 95%CI = 1.67 − 4.01, *p* < 0.001) higher in pregnant women who had the habit consuming raw/unwashed vegetable/meat than those who did not consume raw/unwashed vegetation/meat, and the association in terms of eating soil and raw vegetables is statistically significant (*p* < 0.05) (Table 4).

The multivariate analysis results across sociodemographic, medical history, and potential risk factors are presented as adjusted odds ratio (AOR) at 95% CI in (Tables 2 and 4). The risk factors with *p* < 0.25 in univariate analysis were selected and included in multivariate logistic regression analysis model. As shown in Tables 2 and 4, education level, monthly income, occupation and gravidity, shoes-wearing habit, source of drinking water, and the habit of eating soil and raw vegetables were found to be significant explanatory risk factors of intestinal parasitic infections among the studied pregnant women (*p* < 0.05).

The odds of being infected with IPIs, respectively, were 4.63, 3.64, and 4 times (AOR = 4.63 [1.37-15.66], AOR = 3.64 [1.15-11.54], and AOR = 4.04 [1.26-12.91]) higher in pregnant women who were illiterate, in those who could read and write, and in those who attended high school level than in those who reached college and university education level (*p* < 0.05). Regarding monthly income, the odds of being infected with intestinal parasitic infection were 3.49 times (AOR = 3.49 [1.72-7.07]) higher in pregnant women whose monthly income is less than 500 Birr than those whose monthly income is above 500 ETB (*p* < 0.05). It was also shown that the odds of IPIs were 2 times (AOR = 2.04 [1.01-4.12]) higher in primigravida women than in those multigravidae pregnant women, and this is statistically significant (*p* < 0.05) (Table 2).

In the case of the type of drinking water, the odds of being infected with intestinal parasitic infection were 15 and 7 times (AOR = 14.55 [1.68-126.15], *p* = 0.015, and AOR = 7.02 [0.79-62.41], *p* = 0.080), respectively, higher in pregnant women who drank well/rivers/streams and tap water than in those who drank boiled water. It was also found that the odds of being infected with intestinal parasitic infection were 2.32 times (AOR = 2.32 [1.11-4.87]) higher in pregnant women who ate soil than those who did not eat soil. Similarly, pregnant women who consumed raw/unwashed vegetation/meat were almost 2 times (AOR = 1.91 [1.09-3.31]) more likely to be infected by IPIs than those who did not consume raw/unwashed vegetation/meat, and the association between IPIs and habit of eating soil and raw/unwashed vegetation/meat is statistically significant (*p* < 0.05) (Table 4).

#### 3.3.3. Univariate and Multivariate Analysis of Factors Associated with *E. histolytica/dispar* Infection

Univariate and multivariate logistic regression analysis of factors identified as associated with the risk of *E. histolytica*/*dispar* infection are presented in Table 5. Univariate analysis of risk factors showed that the risk of *E. histolytica/dispar* infection was 5.45, 3.62, 3.46, and 8.83 times (COR = 5.45, 95%CI = 0.71 − 16.74, *p* = 0.124; COR = 3.62, 95%CI = 1.31 − 9.98, *p* = 0.013; COR = 3.46, 95%CI = 1.18 − 10.14, *p* = 0.024; and COR = 8.83, 95%CI = 3.90 − 19.97, *p* ≤ 0.001) higher in pregnant women who were illiterate, in those whose monthly income less than 500 Birr, in those who did not wash hands before meal, and in those who consumed raw vegetables than their reference categories, respectively.

Multivariate analysis also showed that the odds of *E. histolytica*/*dispar* infection were almost 8 times (AOR = 7.83 [3.33-18.41]) higher in pregnant women who consumed raw vegetables/meat than those who did not consume (*p* < 0.05) (Table 5).

#### 3.3.4. Univariate and Multivariate Analysis of Factors Associated with *Ascaris lumbricoides* Infection

The results of univariate and multivariate logistic regression analysis of factors associated with risk of *A. lumbricoides* infections are shown in Table 3. Univariate analysis of risk factors showed that the risk of *A. lumbricoides* infections was 5.47, 4, and 9.93 times (COR = 5.47, 95%CI = 1.46 − 20.58, *p* = 0.012; COR = 4.0, 95%CI = 1.35 − 11.82, *p* = 0.012; and COR = 9.93, 95%CI = 4.51 − 21.87, *p* ≤ 0.001) higher in pregnant women whose monthly income less than 500 Birr, those who did not wash hands before meal and after defecation, than their reference categories, respectively.

Multivariate analysis also showed that the odds of *A. lumbricoides* infection were 4.67 and 8.78 times (4.67 [1.24-17.62] and AOR = 8.78 [3.85-20.01]) higher in pregnant women who did not wash their hands before meal and after defecation than those who washed their hands before meal and after defecation, and it is statistically significant (*p* < 0.05) (Table 3).

## 4. Discussion

The overall prevalence of IPIs among pregnant women in the present study was 36.7%. This is in agreement with the prevalence of 38.7% in Wendo Genet District, southern Ethiopia [28]. On the other hand, the present finding showed a higher prevalence than 29.2% that was reported from Gondar University Hospital [21] and 31.5% reported from Felege Hiwot Referral Hospital, northwest Ethiopia [20]. The high prevalence of IPIs among pregnant women in the study area might be due to low socioeconomic and education status of pregnant women, failing to wear shoes, use of contaminated and unhygienic water, and the habit of eating soil and raw vegetables. However, the present finding's prevalence reports are lower than that from other African countries such as 65% in Gabon [29], 49.6% in Ghana [30], and 76.2% in Kenya [27]. Differences in findings among various studies can be explained by variations in geography, socioeconomic conditions, difference in parasitological examination methods, the number of study population, and the level of awareness about the transmission of IPIs [31].

The overall prevalence of double infection of intestinal parasites in the present study was 1.56%. This finding was comparatively lower than prevalence rates of 3.64% in Felege Hiwot Referral Hospital, northwest Ethiopia [20], 5.36% in Mecha District, northwest Ethiopia [8], and 6.9% report from Nigist Eleni Mohammed Memorial Hospital, southern Ethiopia [32]. The possible differences in findings among studies could be explained by variations in socioeconomic conditions of pregnant women, the methods employed in stool examination, the number of study population, and environmental sanitation [33].

It was found that pregnant women who were illiterate, those who could read and write, and those who attained high school levels of education were 4.63, 3.64, and 4.04 times more likely to be infected with IPIs than those who achieved higher education levels. This finding is in agreement with a report from east Wollega, Oromia Region [34], that showed an increase in the level of educational status of pregnant women having a corresponding lower of risk of parasitic infection. Likewise, the present study also revealed a strong association between monthly income of pregnant women and intestinal parasitic infections. The risk of being infected by intestinal parasite was increased by 3.49 times in pregnant women whose monthly income was less than below 500 ETB than those whose income was greater than 1000 ETB. The finding is in line with a report from Nigist Eleni Mohammed Memorial Hospital [32].

Our study revealed the highest rates of IPIs among primigravida women (41.6%) followed by those who were bigravidae (31.1%) and multigravidae (29.4%). This is in concordance with the findings of Adedojo et al. [35] in Nigeria and Paranjpe et al. [36] in India. It was also shown that the odds of being infected by IPIs were increased by about 2 times among primigravida women than those who were pregnant for multiple times. This result is in line with the findings of studies reported from Entebbe, Uganda [19], Ghana [37], and highlands of Papua New Guinea [38]. This could be explained by the fact that multigravidae women may have benefited from their prior ANC visit of public health education on various aspects of preventive methods and control measures against intestinal parasitic infections.

Pregnant women who used unboiled water from unprotected water sources, well/river/stream for drinking were nearly 15 times more likely to be infected by IPIs than those who were using boiled water. This could be due to contamination of these unprotected water sources by human fecal and animal wastes. This is in agreement with the findings of studies done in Wendo Genet District, southern Ethiopia [28], and Ghana [39] that showed individuals who used unprotected water sources were at increased risk of IPIs.

The present study also demonstrated that the habit of eating soil as one of the significant explanatory risk factors of IPIs. The risk of being infected by intestinal parasites was increased by 2.32 times in pregnant women who ate soil compared with those who did not eat soil during their pregnancy. This study is in agreement with the study conducted from Gilgel Gibe Dam area, southwest Ethiopia [15]. Similarly, pregnant women who consumed raw vegetables were 1.91 times more likely to be infected by intestinal parasitic infections than those who did not consume raw vegetables. Our finding agrees with the study done at Gondar University Hospital [21] that showed uncooked food was significant explanatory risk factor of IPIs.

The habit of consuming raw vegetable was found to be significant explanatory factor of *E. histolytica/dispar* infection. The risk of being infected by *E. histolytica/dispar* was increased by 7.83 times in pregnant women who ingested raw vegetables than those who did not eat raw vegetables. This is in agreement with the finding of Yohannes et al. [21] who also identified ingestion of raw vegetables as significant risk factor of *Entamoeba* infection. In our study, hand washing habit of pregnant women was found to be significant explanatory of *A. lumbricoides* infection. It was found that pregnant women who did not wash their hands before meal and after defecation were 4.67 and 8.78 times more likely to be infected with *A. lumbricoides* infection than those who washed their hands before meal and after defecation, respectively. This finding was in accordance with a study conducted in Mecha District, northwest Ethiopia [8].

This study was limited to only pregnant women who attended Shahura Primary Hospital during the study period. It did not include pregnant women who attended prenatal care in other health centers. Besides, this study employed only direct wet mount and formol-ether concentration methods. The Kato Katz method, which is very important to quantify the loads of parasites, and the Modified Ziehl-Neelsen acid-fast stain, which can be used to detect oocysts of *Cryptosporidium* spp., were not used due to budget constraints.

## 5. Conclusions

The overall prevalence of IPIs among pregnant women attending prenatal care center at Shahura Primary Hospital was substantially higher. The proportion of helminthic infections was higher than that of protozoan infections. *Entamoeba histolytica/dispar*, *G. intestinalis*, *A. lumbricoides*, hookworm, *Taenia* species, *S. stercoralis*, and *S. mansoni* were intestinal parasitic species identified in this study. Education level, monthly income, shoes-wearing habit, source of drinking water, and the habit of eating soil and raw vegetable were significant explanatory variables of IPIs among studied pregnant women in the study area (*p* < 0.05). Therefore, immediate intervention strategies, such as screening of the women for intestinal parasites and provision of health education during their ANC visit and provision of clean and safe drinking water to the community are recommended to prevent the possible adverse maternal and fetal effects resulting from these infections.

## Figures and Tables

**Table 1:** Sociodemographic and obstetric characteristics of pregnant women in Shahura Primary Hospital, 2019 (*n* = 384).

Sociodemographic characteristics	Frequency	Percent
*Age category (year)*		
15-19	20	5.2
20-24	159	41.4
25-29	133	34.6
30-34	33	8.6
35-39	33	8.6
40 and above	6	1.6
*Marital status*		
Single	58	15.1
Divorced	41	10.7
Married	285	74.2
*Education level*		
Illiterate	69	17.9
Writing and reading	174	45.3
High school	93	24.2
College and university	48	12.5
*Residence*		
Rural	188	48.9
Urban	196	51.0
*Monthly income (ETB)*		
Less than 500	80	20.8
500-1000	186	48.4
Above 1000	118	30.7
*Occupation*		
Daily laborer	57	14.8
Student	15	3.9
House wife	268	69.8
Civil servant	44	11.5
*Gravidity*		
Primigravida	214	55.7
Bigravidae	119	31.0
Multigravidae	51	13.3
*Gestation period*		
1st trimester	108	28.1
2nd trimester	174	45.3
3rd trimester	102	26.6

**Table 2:** Univariate and multivariable logistic regression analysis on sociodemographic and obstetric factors associated with IPIs among pregnant women 2019 (*n* = 384).

Sociodemographic variables	Intestinal parasite		COR (95% CI)	*p* value	AOR (95% CI)	*p* value
	Positive (%)	Negative (%)				
*Age category (year)*				*0.734*		
15-19	10 (50.0)	10 (50.0)	5.0 (0.49-50.83)	0.174		
20-24	59 (37.1)	100 (62.9)	2.95 (0.34-25.86)	0.329		
25-29	49 (36.8)	84 (56.4)	2.92 (0.33-25.69)	0.335		
30-34	11 (33.3)	22 (66.7)	2.50 (0.26-24.09)	0.428		
35-39	11 (33.3)	22 (66.7)	2.50 (0.26-24.09)	0.428		
40 and above	1 (16.7)	5 (83.3)	1^∗^			
*Marital status*				*0.572*		
Single	19 (32.8)	39 (67.2)	0.79 (0.43-1.43)	0.432		
Divorced	13 (31.7)	28 (68.3)	0.75 (0.37-1.51)	0.420		
Married	109 (38.2)	176 (61.7)	1^∗^			
*Education level*				*0.004* ^∗^		
Illiterate	34 (49.3)	35 (50.7)	5.69 (2.25-14.42)	≤0.001^∗^	4.63 (1.37-15.66)	0.014^∗^
Writing and reading	64 (36.8)	110 (63.2)	3.41 (1.44-8.04)	0.005^∗^	3.64 (1.15-11.54)	0.028^∗^
High school	36 (38.7)	57 (61.3)	3.69 (1.49-9.13)	0.005^∗^	4.04 (1.26-12.91)	0.019^∗^
College and university	7 (14.6)	41 (85.4)	1^∗^		1^∗^	
*Residence*						
Rural	84 (44.7)	104 (55.3)	1.97 (1.29-3.00)	0.002^∗^	1.56 (0.99-2.47)	0.056
Urban	57 (29.1)	139 (70.9)	1^∗^		1^∗^	
*Monthly income*				*≤0.001* ^∗^		
Less than 500	45 (56.3)	35 (43.7)	3.77 (2.06-6.91)	≤0.001^∗^	3.49 (1.72-7.07)	0.001^∗^
500–1000	66 (35.5)	120 (64.5)	1.61 (0.97-2.69)	0.067	1.48 (0.83-2.64)	0.180
Above 000	30 (25.4)	88 (74.6)	1^∗^		1^∗^	
*Occupation*				*0.019* ^∗^		
Daily laborer	15 (26.3)	42 (73.7)	1.39 (0.54-3.56)	0.494	0.26 (.071-0.95)	0.042^∗^
Student	6 (40.0)	9 (60.0)	2.59 (0.73-9.19)	0.140	0.40 (0.08-2.02)	0.269
House wife	112 (41.8)	157 (58.6)	2.75 (1.27-5.95)	0.010^∗^	0.63 (0.2-1.97)	0.429
Civil servant	9 (20.5)	35 (79.5)	1^∗^		1^∗^	
*Gravidity*				*0.084*		
Primigravida	89 (41.6)	125 (58.4)	1.71 (0.88-3.31)	0.112	2.04 (1.01-4.12)	0.048^∗^
Bigravidae	37 (31.1)	82 (68.9)	1.08 (0.53-2.22)	0.828	1.35 (0.62-2.91)	0.449
Multigravidae	15 (29.4)	36 (70.6)	1^∗^		1^∗^	
*Gestation period*				*0.406*		
1st trimester	43 (39.8)	65 (60.2)	1.45 (0.82-2.56)	0.203		
2nd trimester	66 (37.9)	108 (62.1)	1.34 (0.79-2.25)	0.272		
3rd trimester	32 (31.4)	70 (68.6)	1^∗^			

1^∗^: reference category. ^∗^: statistically significant *p* < 0.05 value.

**Table 3:** Univariate and multivariable logistic regression analysis of selected risk factors associated with *Ascaris lumbricoides* infection among pregnant women.

Characteristic	*Ascaris lumbricoides*		COR (95% CI)	*p* value	AOR (95% CI)	*p* value
	Positive	Negative				
*Monthly income (ETB)*				*0.033*		
Less than 500	10 (12.5)	70 (87.5)	5.47 (1.46-20.58)	0.012^∗^	3.76 (0.88-16.01)	0.075
500-1000	20 (10.8)	166 (89.2)	4.62 (1.34-15.91)	0.015^∗^	2.13 (1.33-20.32)	0.180^∗^
Above 1000	3 (2.5)	115 (97.4)	1^∗^		1^∗^	
*Hand washing before meal*						
No	5 (25.0)	15 (75.0)	4.0 (1.35-11.82)	0.012^∗^	4.67 (1.24-17.62)	0.002^∗^
Yes	28 (7.7)	336 (92.3)	1^∗^		1^∗^	
*Hand washing after toilet*						
No	23 (25.8)	66 (74.1)	9.93 (4.51-21.87)	≤0.001^∗^	8.78 (3.85-20.01)	≤0.001^∗^
Yes	10 (3.4)	285 (96.6)	1^∗^		1^∗^	
*Habit of eating soil*						
Yes	6 (14.6)	35 (85.3)	2.01 (0.77-5.19)	0.151	1.09 (0.34-2.98)	0.994
No	27 (7.9)	316 (92.1)	1^∗^		1^∗^	
*Eating raw vegetables*						
Yes	14 (10.7)	116 (89.2)	1.49 (0.72-3.08)	0.279		
No	19 (7.5)	235 (92.5)	1^∗^			

1^∗^: reference category. ^∗^: statistically significant at *p* < 0.05.

**Table 4:** Univariate and multivariable analysis of potential risk factors associated with IPIs among pregnant women in Shahura Primary Hospital, 2019 (*n* = 384).

Personal hygiene and environmental factors	Intestinal parasite		COR (95% CI)	*p* value	AOR (95% CI)	*p* value
	Positive (%)	Negative (%)				
*Hand washing after toilet*						
No	47 (52.8)	42 (47.2)	2.39 (1.48-3.88)	≤0.001^∗^	1.34 (0.73-2.48)	0.345
Yes	94 (31.9)	201 (68.1)	1^∗^		1^∗^	
*Hand washing before meal*						
No	14 (70.0)	6 (30.0)	4.35 (1.63-11.60)	0.003^∗^	2.47 (0.84-7.31)	0.102
Yes	127 (34.9)	237 (65.1)	1^∗^		1^∗^	
*Place of defecation*						
Outside latrine	56 (45.2)	68 (54.8)	1.69 (1.09-2.63)	0.018^∗^	1.16 (0.70-1.91)	0.568
Inside latrine	85 (32.7)	175 (67.3)	1^∗^		1^∗^	
*Place of bath*						
Outside house (river)	52 (44.1)	66 (55.9)	1.57 (1.01-2.44)	0.047^∗^	0.92 (0.54-1.56)	0.749
Inside house	89 (33.5)	177 (66.5)	1^∗^		1^∗^	
*Habit of wearing shoe*						
No	28 (58.3)	20 (41.7)	2.76 (1.49-5.12)	≤0.001^∗^	0.72 (1.36-5.43)	0.005^∗^
Yes	113 (33.6)	223 (66.4)	1^∗^		1^∗^	
*Type of drinking water*						
Well/stream/river (unboiled)	76 (51.4)	72 (48.6)	9.50 (1.17-76.88)	0.035^∗^	14.55 (1.68-126.15)	0.015^∗^
Tap water	64 (28.3)	162 (71.7)	3.56 (0.44-28.64)	0.233	7.02 (0.79-62.41)	0.080
Boiled water	1 (10.0)	9 (90.0)	1^∗^		1^∗^	
*Soil eating habit*						
Yes	24 (58.5)	17 (41.5)	2.73 (1.41-5.28)	0.003^∗^	2.32 (1.11-4.87)	0.026^∗^
No	117 (34.1)	226 (65.8)	1^∗^		1^∗^	
*Habit of eating raw vegetable/meat*						
Yes	67 (51.5)	63 (48.5)	2.58 (1.67-4.01)	≤0.001^∗^	1.91 (1.09-3.31)	0.023^∗^
No	74 (29.1)	180 (70.8)	1^∗^		1^∗^	

1^∗^: reference category; ^∗^: statistically significant at *p* < 0.05.

**Table 5:** Univariate and multivariable logistic regression analysis of selected risk factors associated with *E. histolytica/dispar* infections among pregnant women.

Characteristic	*Entamoeba histolytica*		COR (95% CI)	*p* value	AOR (95% CI)	*p* value
	Positive	Negative				
*Educational level*				*0.081*		
Illiterate	9 (13.1)	60 (86.9)	5.45 (0.71-16.74)	0.124	2.33 (0.42-13.11)	0.336
Writing and reading	22 (12.6)	152 (87.3)	3.33 (0.75-14.69)	0.112	3.79 (0.76-18.56)	0.099
High school	4 (4.3)	89 (95.7)	1.03 (0.18-5.86)	0.970	1.19 (0.19-7.34)	0.853
College/university	2 (4.2)	46 (95.8)	1^∗^		1^∗^	
*Monthly income (ETB)*				*0.041*		
Less than 500	13 (16.2)	67 (83.7)	3.62 (1.31-9.98)	0.013^∗^	1.69 (0.53-5.34)	0.373
500-1000	18 (9.7)	168 (90.3)	2.0 (0.77-5.19)	0.155	1.58 (0.54-4.64)	0.397
Above 1000	6 (5.1)	112 (94.9)	1^∗^		1^∗^	
*Hand washing before meal*						
No	5 (25.0)	15 (75.0)	3.46 (1.18-10.14)	0.024^∗^	2.81 (0.80-9.83)	0.106
Yes	32 (8.8)	332 (91.2)	1^∗^		1^∗^	
*Place of defecation*						
Outside toilet	15 (12.1)	109 (87.9)	1.49 (0.74-2.98)	0.261		
Inside toilet	22 (8.5)	238 (91.5)	1^∗^			
*Habit of eating raw vegetables/meat*						
Yes	29 (22.3)	101 (77.7)	8.83 (3.90-19.97)	≤0.001^∗^	7.83 (3.33-18.41)	≤0.001^∗^
No	8 (3.1)	206 (96.3)	1^∗^		1^∗^	

1^∗^: reference category. ^∗^: statistically significant at *p* < 0.05.

## Data Availability

The datasets used and/or analyzed during the current study are available from the corresponding author upon reasonable request.
